# Non-coding RNA biomarkers in resistant hypertension: a scoping review

**DOI:** 10.3389/fmolb.2026.1786399

**Published:** 2026-03-12

**Authors:** Robert Błaszczyk, Maciej Biskupski, Łukasz Wiśniowski, Bartosz Kondracki, Jan Siwiec, Radosław Mlak, Andrzej Głowniak

**Affiliations:** 1 Department of Cardiology, Medical University of Lublin, Lublin, Poland; 2 Department of Plastic, Reconstructive Surgery and Microsurgery, Medical University of Lublin, Lublin, Poland; 3 Department of Pneumonology, Oncology and Allergology, Medical University of Lublin, Lublin, Poland; 4 Department of Laboratory Diagnostics, Medical University of Lublin, Lublin, Poland

**Keywords:** biomarkers, microRNA, noncoding RNA, obstructive sleep apnea, resistant hypertension

## Abstract

**Objective:**

To map and synthesize human evidence on circulating non-coding RNAs (ncRNAs) evaluated as biomarkers in resistant hypertension (RH), including associations with RH phenotypes, target-organ damage, and short-term treatment response.

**Methods:**

A scoping review was conducted and reported in line with PRISMA-ScR guidance. PubMed, Web of Science, and Google Scholar were searched using RH-related terms combined with ncRNA keywords. After deduplication, titles/abstracts and full texts were screened in Covidence. Eligible records included full-length articles and conference abstracts reporting original human research explicitly addressing RH; case reports, narrative reviews, and animal/in vitro-only studies were excluded. Data were charted using a standardized extraction form and synthesized qualitatively due to heterogeneity.

**Results:**

Eight studies met inclusion criteria (five full-length articles and three congress abstracts) published between 2013 and 2024. Across studies, RH sample sizes ranged from 10 to 115 (total RH participants: 338; mean ≈ 42 per study). All studies assessed peripheral-blood matrices (serum, plasma, or PBMCs) and focused on microRNAs (miRNAs) or circular RNAs (circRNAs); no eligible study evaluated other circulating ncRNA classes (e.g., lncRNAs, snoRNAs, siRNAs, piRNAs) specifically in RH. Evidence clustered into four clinical contexts: baseline RH comparisons (and separately, internal comparison of RH with versus without type 2 diabetes mellitus), RH with obstructive sleep apnoea (OSA) and blood-pressure response to CPAP, RH with and without OSA, and RH treated with renal sympathetic denervation (RDN). Reported signals included elevated serum miR-21 in RH versus comparators (AUC ≈0.82 in one study), higher PBMC miR-1-1 and miR-195 in RH with diabetes, a three-miRNA panel (miR-378a-3p/miR-486-5p/miR-100-5p) discriminating CPAP responders versus non-responders (AUCs ≈0.88 training and ≈0.92 validation), increased miR-133a after RDN (≈7.2-fold), and early post-RDN circRNA changes (hsa_circRNA_000367↑; hsa_circRNA_405119↓). Methodology (platforms and normalization) and analytic rigor varied substantially; most analyses were univariable and external validation was absent.

**Conclusion:**

Human evidence on circulating ncRNA biomarkers in RH is sparse and fragmented. Although individual studies have reported biologically plausible associations between selected miRNAs/circRNAs and RH-related phenotypes or short-term treatment response, current data are insufficient for clinical implementation. Larger, well-phenotyped, multicentre studies using standardized assays and independent, robust multivariable validation are needed.

## 1 Introduction

Arterial hypertension remains one of the leading, modifiable contributors to cardiovascular morbidity and mortality worldwide. The most recent global analyses estimate that the number of adults aged 30–79 years with hypertension has doubled since 1990, reaching more than 1.2 billion people in 2019, with especially rapid growth in low- and middle-income countries. Despite stable age-standardised prevalence, only about 50% of individuals with hypertension receive treatment and fewer than one in four achieve adequate blood pressure (BP) control (World Health Organization, 2023; NCD Risk Factor Collaboration, 2021; Pathak et al., 2022). Uncontrolled hypertension substantially increases the risk of stroke, myocardial infarction, heart failure, chronic kidney disease and premature death, and has been recognized by the World Health Organization as a “silent killer” with major societal and economic impact (World Health Organization, 2023).

Within this global context, resistant hypertension (RH) represents a particularly high-risk phenotype. Contemporary guidelines of the American Heart Association define RH as office BP remaining above target despite the concurrent use of three antihypertensive drug classes (including a diuretic) at maximally tolerated doses, or as controlled BP requiring four or more agents (Carey et al., 2018). A large meta-analysis including over 3.2 million treated hypertensive patients estimated the prevalence of apparent treatment-resistant hypertension at about 15% in the general hypertensive population, with higher proportions among individuals with chronic kidney disease, renal transplant or advanced age (Noubiap et al., 2019). After exclusion of pseudo-resistance related to white-coat effect, inadequate measurement or poor adherence, true RH still affects roughly 10% of treated patients (Noubiap et al., 2019). Individuals with RH have a longer history of hypertension and a greater burden of obesity, diabetes, left ventricular hypertrophy, albuminuria and renal dysfunction than those with controlled hypertension, translating into a markedly increased risk of cardiovascular events and target-organ damage (Oliveras and de la Sierra, 2014).

The mechanisms underlying RH are multifactorial and incompletely understood. Current concepts emphasize an interplay between persistent activation of the renin–angiotensin–aldosterone system (RAAS), heightened sympathetic nervous system activity, renal sodium retention, vascular stiffness, endothelial dysfunction and structural cardiac remodeling (Carey et al., 2018; Faselis et al., 2011). In many patients, RH coexists with secondary contributors such as primary aldosteronism, renal parenchymal disease, renal artery stenosis, obstructive sleep apnoea and high dietary sodium intake (Carey et al., 2018; Faselis et al., 2011). Distinguishing true RH from pseudo-resistance requires standardized BP measurement, out-of-office monitoring, careful assessment of adherence and exclusion of interfering substances (Champigny and Nagge, 2024).

Therapeutic management of RH is correspondingly complex. Guideline-recommended strategies include optimization of lifestyle measures, standardized combination therapy (typically a renin–angiotensin system blocker, a calcium-channel blocker and a thiazide-like diuretic), systematic screening for secondary causes and the addition of a mineralocorticoid-receptor antagonist, which provides substantial incremental BP reduction in many patients (Faconti et al., 2025). For selected patients with true RH despite optimal pharmacotherapy, interventions such as catheter-based renal sympathetic denervation (RDN) have re-emerged as potential adjunctive treatments, with recent trials demonstrating modest but clinically relevant reductions in office and ambulatory BP (Faconti et al., 2025). Nonetheless, response to any given therapeutic strategy is heterogeneous, and robust tools for predicting which patients will benefit from specific interventions are lacking. This has stimulated growing interest in molecular and epigenetic biomarkers that might refine risk stratification and guide individualized treatment.

Over the past decade, non-coding RNAs (ncRNAs)—and in particular microRNAs (miRNAs)—have emerged as important regulators of gene expression in cardiovascular disease. MiRNAs are short, non-coding RNA molecules that bind to target messenger RNAs and modulate their stability or translation, thereby influencing whole networks of genes rather than single pathways (de Gonzalo-Calvo et al., 2017). A growing body of experimental and clinical work indicates that altered ncRNA profiles are involved in the initiation and progression of hypertension and its target-organ complications, and that changes in specific ncRNAs can be linked to blood pressure levels, vascular and cardiac remodeling, and associated cardiovascular risk (de Gonzalo-Calvo et al., 2017; Shaheen et al., 2023). Importantly, ncRNAs are not confined to intracellular compartments. MiRNAs and circRNAs are released into the circulation, where they are stabilized by encapsulation in extracellular vesicles, association with lipoproteins or binding to Argonaute proteins (Zhu and Fan, 2011). These circulating ncRNAs are detectable with high analytical sensitivity and exhibit disease- and tissue-specific expression patterns, making them attractive candidates as minimally invasive biomarkers and, potentially, as mediators of inter-organ communication (Zhu and Fan, 2011).

The aim of this scoping review is therefore to identify, describe and synthesize human studies that have evaluated ncRNAs biomarkers in the context of RH. Specifically, the review will map (1) which RNA species have been studied, in which biological matrices and clinical phenotypes of RH; (2) how RH was defined and which comparators were used in these studies; and (3) the reported associations between these RNA markers and blood pressure levels, aldosterone and other neurohormonal parameters, measures of cardiac or vascular remodeling, and short-term treatment response. By charting the spectrum of RNA targets, study designs, analytical platforms and clinical correlations, this review aims to clarify the current state of evidence and to highlight key methodological limitations and gaps that should inform the design of future translational and clinical studies on RNA biomarkers in RH.

## 2 Methods

**FIGURE 1:**
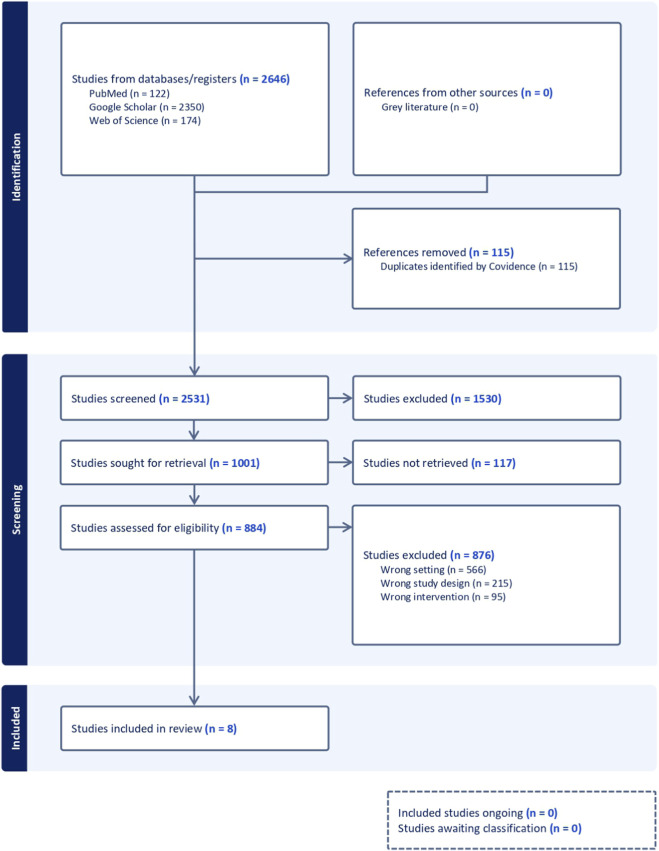
PRISMA-style flow diagram of study identification, screening, eligibility assessment and inclusion. Database/register searching identified 2,646 records (PubMed, n = 122; Web of Science, n = 174; Google Scholar, n = 2,350); no records were identified from other sources (n = 0). After removal of duplicates (n = 115; identified by Covidence), 2,531 records were screened, of which 1,530 were excluded. A total of 1,001 reports were sought for retrieval; 117 were not retrieved, leaving 884 reports assessed for eligibility. Of these, 876 were excluded (wrong setting, n = 566; wrong study design, n = 215; wrong intervention, n = 95). Eight studies were included in the review (n = 8), including three conference abstracts (n = 3).

This scoping review was conducted to map and synthesize human studies that evaluated ncRNA biomarkers in the context of resistant or uncontrolled hypertension. The review was conducted in line with methodological guidance for scoping reviews and reported according to the Preferred Reporting Items for Systematic Reviews and Meta-Analyses extension for Scoping Reviews (PRISMA-ScR) (Tricco et al., 2018). The study selection process is presented in Figure 1 using a PRISMA flow diagram adapted from the PRISMA 2020 template (Page et al., 2021). Comprehensive literature searches were performed in PubMed, Web of Science and Google Scholar, with the final search update conducted on 15 December 2025. No restrictions on year of publication were applied. Searches were limited to English-language records; no additional database filters were applied at the database-search stage. RH-related terms were combined with a comprehensive ncRNA keyword set, and the same search string was used in PubMed and Web of Science: (microRNA* OR “micro RNA*” OR miRNA* OR “miR*” OR “miR-” OR “long noncoding RNA” OR “long non-coding RNA*” OR lncRNA* OR “lnc RNA*” OR “circular RNA*” OR circRNA* OR “noncoding RNA*” OR “non-coding RNA*” OR ncRNA* OR “small nucleolar RNA*” OR snoRNA* OR “small interfering RNA*” OR siRNA* OR “PIWI-interacting RNA*” OR piRNA* OR “messenger RNA*” OR mRNA*) AND (“resistant hypertension” OR “treatment-resistant hypertension” OR “treatment resistant hypertension” OR “refractory hypertension”). Google Scholar was used as a supplementary source because the RH ncRNA biomarker literature is sparse and some early findings may be disseminated outside traditional indexing; however, Google Scholar is imprecise and has limited reproducibility. To mitigate this, we used a simplified concept-consistent query and screened results sorted by relevance, restricting screening to the first 500 records. All records were imported into Covidence where possible and duplicates were removed prior to screening. Titles/abstracts and full texts were screened in Covidence by two reviewers working independently at each stage. Disagreements were resolved by discussion; if consensus could not be reached, a third reviewer adjudicated. Formal inter-reviewer agreement statistics were not calculated. Eligible records included original human research explicitly addressing RH and reporting circulating or peripheral-blood ncRNAs in relation to RH phenotypes, target-organ damage, or short-term treatment response. Full-length peer-reviewed articles and conference abstracts were eligible; abstracts were included *a priori* to map emerging evidence in this nascent field, but were treated as preliminary/hypothesis-generating because they typically provide limited methodological detail. We excluded case reports, narrative reviews, animal-only or in vitro-only studies, and studies focused solely on genetic predisposition to primary hypertension without explicit reference to RH. Data were charted using a standardized extraction form capturing study characteristics, definition of RH, type of RNA, biological matrix, analytical methods, main findings and limitations. Given the heterogeneity of study designs and outcomes, a qualitative synthesis was undertaken to identify recurring themes and patterns across studies, and no quantitative meta-analysis was performed.

We anticipated heterogeneity in how RH was operationalized across primary studies and therefore extracted and reported the exact RH definition used by each study (including blood-pressure thresholds, number/classes of antihypertensive drugs, and whether out-of-office confirmation and work-up for pseudo-resistance were described). For eligibility, we accepted studies in which participants were explicitly described as having resistant/treatment-resistant/refractory hypertension, or met a drug-based definition consistent with RH (uncontrolled BP on ≥3 antihypertensive classes including a diuretic, or controlled BP requiring ≥4 agents), even when full details of adherence assessment or exclusion of secondary causes were not reported.

Consistent with the objectives of a scoping review, we did not apply a formal risk-of-bias tool across studies, because included records were methodologically heterogeneous (cross-sectional biomarker comparisons, pre–post interventional cohorts, and abstract-only reports) and many did not report the information required for domain-level judgments using standard tools (e.g., QUADAS-2/ROBINS-I). Instead, key bias-relevant methodological features were charted *a priori* for each record and summarised in Table 1, including cohort size, RH phenotyping rigor (true RH vs. apparent/incompletely phenotyped RH), assay and normalization reporting/standardisation, statistical adjustment for confounders and multiple testing, validation/replication (technical, internal, or external), and publication type (full article vs. conference abstract).

**TABLE 1:** Characteristics and key findings of studies investigating RNA biomarkers in resistant hypertension.

Study	Evidence level	Study design	Matrix	RH definition	Comparator(s)	Sample size (RH total)	Platform	Normalization	Biomarkers	Key findings	Key limitations/notes
Kara et al. (2021)	Full article	Cross-sectional case-control	Serum	>=3 drugs incl diuretic (max doses); pseudo-resistance excluded; secondary causes excluded; ABPM ≥130/80 mmHg	Normotensive; newly diagnosed untreated essential HT	20	Targeted TaqMan; ddPCR	ddPCR absolute copies/uL; Poisson normalization; reference miRNA not specified	miR-21; miR-155	miR-21 higher in RH vs. both comparators; cutoff ∼9.6 copies/uL; AUC ∼0.82; miR-155: no group difference; adj: age + BMI (ANCOVA)	Single-centre; modest N; RH older/comorbid; heavy multidrug therapy; no external validation
Sánchez-de-la-Torre et al. (2015)	Full article	Multicentre RCT (open-label CPAP); biomarker substudy; men with RH + OSA	Plasma	ABPM ≥130/80 on ≥3 drugs (ideally incl diuretic); adherence verified; major secondary causes excluded	CPAP responders vs. non-responders (≥4.5 mmHg fall in 24-h mean BP; 3 months)	38 (20 responders; 18 non-responders)	Qiagen 84-miRNA PCR array; qRT-PCR validation	SNORD95; dCt/ddCt	84-miRNA panel; selected 8 (miR-7-5p, miR-29a-3p, miR-92a-3p, miR-100-5p, miR-144-3p, miR-150-5p, miR-378a-3p, miR-486-5p)	Internal logistic model for BP response: miR-378a-3p/miR-486-5p/miR-100-5p; AUC ∼0.88 (training) and ∼0.92 (validation); no external replication; no non-RH comparator	Men only; small N; internal (not external) validation; clinical covariate adjustment limited/unclear; blood-sampling refusals reported
Schirmer et al. (2013)	Congress abstract	Prospective pre-post cohort after RDN	Plasma	Described as RH; formal definition/BP cut-offs not reported	Pre vs. 6 months post-RDN (no non-RDN control)	40 (20 paired discovery; +20 validation cohort)	Agilent 8 × 60K *Homo sapiens* microRNA v16 microarrays; qRT-PCR validation	Quantile normalization; FDR<5%; qRT-PCR reference not reported	Post-RDN miRNAs (34 reported; IDs not listed)	BP decreased after RDN; selected miRNA changes correlated with BP reduction and LV mass index regression (miRNA IDs not provided)	Abstract-only; small cohorts; no control; RH criteria not reported; miRNA IDs not listed; no covariate adjustment reported
Dörr et al. (2016)	Full article	Prospective pre-post cohort after RDN (6-month follow-up)	Serum	ESC/ESH-aligned RH; office SBP >140 on ≥3 drugs incl diuretic; ABPM threshold used; secondary causes excluded; stable regimen	Pre vs. 6 months post-RDN (no external control)	90	Targeted qRT-PCR (TaqMan assays)	Cel-miR-39 spike-in; triplicates	miR-133a; miR-21	At 6 months: office SBP and 24-h SBP decreased (both p < 0.001); miR-133a increased (∼7.2-fold; p = 0.003); miR-21: no significant change	Non-randomized; no control; univariable analyses only; potential adherence/Hawthorne effects; miRNA change may reflect BP lowering rather than RDN-specific biology
Błaszczyk et al. (2024)	Full article	Cross-sectional; within-RH subgroup comparison	PBMCs	ESH-aligned RH; triple therapy (RAAS blocker + long-acting CCB + thiazide/thiazide-like diuretic) at max tolerated doses; ABPM/HBPM confirmation; pseudo-resistance/secondary causes excluded	RH with T2DM vs. RH without T2DM	115 (53 with T2DM; 62 without)	Targeted TaqMan qRT-PCR panel	RNU48 (SNORD48); ddCt; logRQ	Panel incl miR-1-1, miR-21, miR-26b, miR-126, miR-133a, miR-143, miR-145, miR-155, miR-195, miR-208, miR-320	RH + T2DM: miR-1-1 and miR-195 higher vs. non-diabetic RH; trends toward higher miR-126, miR-133a, miR-143 and miR-320a in the T2DM group; no significant differences for miR-145, miR-155, miR-21, miR-26b or miR-208a	Single-centre; PBMC matrix; all participants RH (limits RH-specific inference); univariable analyses; risk of false positives with multiple comparisons; highly comorbid
Cai et al. (2022)	Full article	Prospective pre-post cohort after RDN (48 h; 12 months follow-up reported)	Serum	Chinese 2018 guideline RH; uncontrolled on ≥3 (incl diuretic) or controlled on ≥4; office ≥140/90; home ≥135/85; 24-h mean ≥130/80 (pre-RDN)	Pre vs. 48 h post-RDN (no controls)	13 (5 profiled by array; 13 qPCR validation)	Arraystar 8 × 15K human circRNA array v2 (RNase R); RT-qPCR validation	Quantile normalization; FC>=1.2 and p < 0.05; GAPDH (RT-qPCR)	CircRNAs (338 differentially expressed); validated: hsa_circRNA_000367 (circSIAE); hsa_circRNA_405119	48 h post-RDN: hsa_circRNA_000367 up; hsa_circRNA_405119 down; 12-month home SBP decreased by ∼12 mmHg	Very small; single-centre; no controls; acute 48-h profiling; limited linkage to clinical endpoints; univariable tests
Novak et al. (2017)	Congress abstract	Pilot cross-sectional cohort (RH + habitual snoring)	Peripheral blood (matrix not specified)	Described as RH; formal criteria/BP thresholds not reported	RH + OSA vs. RH without OSA	10 (8 with OSA; 2 without)	Targeted RT-qPCR (3 miRNAs)	Endogenous control/normalization not reported	miR-210; miR-126; miR-499	miR-210 and miR-126 higher in RH + OSA vs. RH without OSA (p = 0.045); miR-499: NS; no correlations reported	Abstract-only; very small; only 2 without OSA; RH definition and regimen not reported; univariable tests only; no non-RH comparator
Khalyfa et al. (2015)	Congress abstract	Prospective pilot (CPAP in RH + OSA); responder vs. non-responder comparison	Plasma	Described as RH; formal criteria/BP thresholds not reported	CPAP responders vs. non-responders (3 months)	12 (6 responders; 6 non-responders; men)	Qiagen 84-miRNA array (baseline and 3 months CPAP)	Endogenous control/normalization not reported	84-miRNA cardiovascular panel (miRNA identities not listed)	Baseline: 7 miRNAs differed between responder/non-responder groups; post-CPAP: 46 miRNAs changed in responders (reported); miRNA identities not provided; exploratory	Abstract-only; small; men only; no non-RH/non-OSA controls; limited reporting (miRNA IDs, normalization, covariate adjustment); needs full publication/validation

## 3 Results

Eight original human studies met the inclusion criteria, comprising five full-length peer-reviewed articles and three congress abstracts, published between 2013 and 2024. The three congress abstracts (Schirmer et al., 2013; Novak et al., 2017; Khalyfa et al., 2015) were considered low-level evidence because reporting is typically incomplete and peer review is limited; therefore, they were used to map emerging signals rather than to support firm conclusions about specific biomarkers. These investigations evaluated circulating RNA biomarkers in patients with RH across four main clinical contexts: (1) baseline RH compared with normotension and newly diagnosed essential hypertension (Kara et al., 2021) and, separately, internal comparison of RH with versus without type 2 diabetes mellitus (T2DM) (Błaszczyk et al., 2024); (2) RH with concomitant obstructive sleep apnoea (OSA) and BP response to continuous positive airway pressure (CPAP) (Sánchez-de-la-Torre et al., 2015; Khalyfa et al., 2015); (3) RH with and without OSA in a small screening cohort (Novak et al., 2017); and (4) RH treated with catheter-based renal sympathetic denervation (RDN) (Schirmer et al., 2013; Dörr et al., 2016; Cai et al., 2022). All studies used peripheral blood as the source material—serum, plasma, or peripheral blood mononuclear cells (PBMCs)—and focused on miRNAs or circRNAs; no eligible study assessed other circulating RNA classes such as lncRNAs, snoRNAs, siRNAs or piRNAs specifically in RH. Table 1 summarises the characteristics and key findings of studies investigating RNA biomarkers in RH.

Across the eight studies, the number of patients with RH per study ranged from 10 to 115, with a mean of approximately 42 RH participants per study (338 individuals in total). In most reports, RH was defined in line with contemporary guidelines—requiring uncontrolled office or ambulatory blood pressure despite at least three antihypertensive drug classes, including a diuretic, with exclusion of secondary causes and pseudo-resistance—although some abstracts did not specify formal cut-offs or adherence assessment (Schirmer et al., 2013; Novak et al., 2017; Khalyfa et al., 2015). The populations were typically middle-aged or older, with a high burden of long-standing hypertension, obesity, diabetes and target-organ damage.

Study designs and laboratory approaches were heterogeneous. Two studies used cross-sectional observational designs with internal comparison groups (Kara et al., 2021; Błaszczyk et al., 2024); one randomized controlled trial embedded a biomarker substudy (Sánchez-de-la-Torre et al., 2015); two prospective pre–post cohorts evaluated RNA changes after RDN (Dörr et al., 2016; Cai et al., 2022); one microarray-based RDN study included a small discovery and validation cohort (Schirmer et al., 2013); and two pilot OSA-related studies were exploratory observational cohorts (Novak et al., 2017; Khalyfa et al., 2015). Platforms ranged from targeted TaqMan assays and droplet digital PCR (ddPCR) for individual miRNAs (Kara et al., 2021; Dörr et al., 2016; Błaszczyk et al., 2024) to 84-miRNA cardiovascular arrays (Sánchez-de-la-Torre et al., 2015; Khalyfa et al., 2015), whole-genome miRNA microarrays (Schirmer et al., 2013) and circRNA microarrays (Cai et al., 2022). Normalisation strategies varied widely, including absolute copy number estimation by ddPCR without a single housekeeping miRNA (Kara et al., 2021), endogenous controls such as SNORD48 (RNU48) or SNORD95 (Sánchez-de-la-Torre et al., 2015; Błaszczyk et al., 2024), exogenous spike-in cel-miR-39 (Dörr et al., 2016), GAPDH for circRNA RT-qPCR (Cai et al., 2022), and, in some abstracts, unspecified internal controls (Novak et al., 2017; Khalyfa et al., 2015). Statistical analyses were predominantly univariable (group comparisons and simple correlations); only one study used multivariable logistic regression to derive and internally validate a predictive miRNA model (Sánchez-de-la-Torre et al., 2015).

### 3.1 miRNAs in baseline resistant hypertension

Two full-length studies directly evaluated circulating miRNAs in well-phenotyped RH cohorts. Kara et al. (2021) compared serum miR-21 and miR-155 among 20 patients with RH, 30 patients with newly diagnosed untreated essential hypertension and 32 normotensive controls. RH was stringently defined: BP above target despite ≥3 antihypertensive drugs (including a diuretic) at maximal doses, exclusion of pseudo-resistance and secondary causes, and confirmation of uncontrolled BP by 24-h ambulatory monitoring (≥130/80 mmHg). Using ddPCR, the authors found that miR-21 concentrations were markedly higher in RH than in both newly diagnosed hypertension and normotension, whereas miR-155 showed no significant between-group differences (Kara et al., 2021). A miR-21 threshold of approximately 9.6 copies/µL discriminated RH from non-resistant status with an area under the ROC curve of about 0.82, high sensitivity and moderate specificity. Serum miR-21 correlated positively with aldosterone, age, office and 24-h systolic BP, fasting glucose and albuminuria, suggesting that it captures a composite signal of RAAS activation, haemodynamic load and early renal damage in RH (Kara et al., 2021).

Błaszczyk et al. (2024) analysed PBMC expression of a predefined panel of cardiovascular miRNAs in 115 patients with RH, comparing those with (n = 53) and without (n = 62) T2DM. RH was diagnosed according to European Society of Hypertension (ESH) criteria and confirmed by ambulatory or home blood pressure monitoring, with careful exclusion of pseudo-resistance and secondary causes (Błaszczyk et al., 2024). Among the 11 miRNAs assessed (including miR-1-1, miR-21, miR-26b, miR-126, miR-133a, miR-143, miR-145, miR-155, miR-195, miR-208 and miR-320), expression of miR-1-1 and miR-195 was significantly higher in patients with RH plus T2DM than in those without T2DM, with additional non-significant trends towards higher miR-126, miR-133a, miR-143 and miR-320a in the diabetic group. No significant differences were observed for miR-145, miR-155, miR-21, miR-26b or miR-208 (Błaszczyk et al., 2024). Patients with T2DM had more advanced structural heart disease and chronic kidney disease, including larger left atrial dimensions, more left ventricular hypertrophy, higher prevalence of heart failure with preserved ejection fraction and more frequent prior coronary revascularisation. In the subgroup without T2DM, longer duration of RH correlated negatively with miR-145 and miR-208a expression (Błaszczyk et al., 2024). These findings imply that selected miRNAs—particularly miR-1-1 and miR-195—may reflect the added cardiometabolic burden and remodelling associated with T2DM in RH, rather than susceptibility to resistance itself.

Taken together, Kara and Błaszczyk provide complementary perspectives: miR-21 appears to differentiate RH from less advanced hypertensive and normotensive states, whereas broader PBMC miRNA profiles help stratify risk within the RH population according to metabolic status and organ-damage burden (Kara et al., 2021; Błaszczyk et al., 2024). Both studies, however, are single-centre, rely largely on univariable analyses and lack external validation cohorts.

### 3.2 miRNA signatures in resistant hypertension with obstructive sleep apnoea

Three studies investigated circulating miRNAs in patients with RH and OSA. In a multicentre randomized trial of men with RH and moderate-to-severe OSA, Sánchez-de-la-Torre et al. (2015) measured an 84-miRNA cardiovascular panel in plasma at baseline and after 3 months of CPAP. RH was rigorously defined using 24-h ambulatory blood pressure monitoring (mean systolic ≥130 mmHg and/or diastolic ≥80 mmHg) despite at least three antihypertensive drugs, with adherence verified and major secondary causes excluded. Among 38 patients (20 CPAP responders, defined as a reduction in mean 24-h blood pressure >4.5 mmHg, and 18 non-responders), the authors identified a three-miRNA cluster (miR-378a-3p, miR-486-5p and miR-100-5p) whose baseline expression discriminated responders from non-responders with high accuracy, yielding AUCs around 0.88 in the training set and 0.92 in the validation set. In responders, CPAP induced broad downregulation of cardiovascular miRNAs and a greater reduction in the aldosterone-to-renin ratio than in non-responders; changes in aldosterone/renin correlated with blood-pressure reductions (Sánchez-de-la-Torre et al., 2015).

Two smaller pilot studies, both available only as conference abstracts, provided additional but less detailed data. Novak et al. (2017) examined 10 patients with RH and habitual snoring (8 with polysomnography-confirmed OSA and 2 without OSA) and reported higher relative expression of miR-210 and miR-126 in the OSA subgroup, without robust correlations with BP or apnoea–hypopnoea index. Khalyfa et al. (2015) profiled 84 cardiovascular miRNAs in 12 men with RH and OSA (n = 6 responders; n = 6 non-responders) and reported baseline differences and post-CPAP expression shifts, but did not provide specific miRNA identities or multivariable modelling. Taken together, the OSA-focused evidence remains preliminary and largely hypothesis-generating, given very small sample sizes, internal validation, incomplete reporting in abstract-only studies, and limited adjustment for confounding.

### 3.3 Circulating RNA changes after renal sympathetic denervation

Three studies evaluated the impact of RDN on circulating RNA profiles in RH. Schirmer et al. (2013) performed whole-genome miRNA microarrays in plasma from 20 patients with RH before and 6 months after RDN, with validation of selected miRNAs in an independent cohort of 20 additional patients. Although a formal definition of RH was not provided in the abstract, all participants were described as having RH undergoing catheter-based RDN. The authors reported 34 miRNAs as differentially expressed after RDN (26 upregulated and 3 downregulated, with the remaining differentially expressed miRNAs not clearly categorised in the abstract) and observed that post-to-pre procedural ratios of several miRNAs correlated with changes in echocardiographic indices, including left ventricular mass index, longitudinal strain and left atrial volume index (Schirmer et al., 2013). These associations suggest that miRNA dynamics may reflect early cardiac reverse-remodelling processes in RDN-treated RH, but the absence of detailed miRNA identities and multivariable analyses limits interpretability.

Dörr et al. (2016) studied 90 patients with RH defined according to ESC/ESH criteria and Symplicity HTN trial inclusion thresholds (office systolic BP > 160 mmHg or >150 mmHg in T2DM, and/or elevated daytime ambulatory systolic BP). Serum miR-133a and miR-21 were quantified before and 6 months after RDN using qRT-PCR with an exogenous cel-miR-39 spike-in for normalisation. At 6 months, office systolic BP fell by about 21 mmHg and 24-h ambulatory systolic BP by approximately 15 mmHg (both p < 0.001), while serum miR-133a increased by a mean 7.2-fold (p = 0.003) and miR-21 showed no significant change. Patients with lower baseline miR-133a experienced greater increases in miR-133a and slightly larger BP reductions than those with higher baseline values; baseline miR-133a correlated inversely with its fold change (r = −0.59), and baseline systolic BP correlated inversely with blood-pressure reduction (r = −0.47). Left-ventricular mass indices and natriuretic peptide levels did not change significantly over 6 months (Dörr et al., 2016). The authors interpreted the rise in circulating miR-133a as a potential early indicator of reverse remodelling in hypertensive heart disease, particularly in those with an initially unfavourable miRNA profile.

Cai et al. (2022) investigated serum circRNA profiles in 13 patients with RH undergoing RDN. RH was defined according to 2018 Chinese guidelines, requiring uncontrolled office, home and 24-h BP despite at least three antihypertensive drugs including a diuretic, or the need for ≥4 drug classes to achieve control. Five paired samples were used for discovery on an Arraystar human circRNA microarray after RNase R enrichment, and all 13 paired samples were used for RT-qPCR validation of selected circRNAs. Within 48 h of RDN, 338 circRNAs were differentially expressed (170 up- and 168 downregulated), and RT-qPCR confirmed significant upregulation of hsa_circRNA_000367 and downregulation of hsa_circRNA_405119 (Cai et al., 2022). Gene-ontology and pathway analyses of predicted circRNA–miRNA–mRNA networks suggested involvement of inflammatory, immune, complement/coagulation, cytoskeletal and focal-adhesion pathways potentially related to sympathetic nerve injury and vascular responses after RDN. At 12-month follow-up, home systolic BP had fallen by about 12 mmHg on average, but the study did not report direct correlations between individual circRNA changes and BP or organ-damage indices (Cai et al., 2022).

Overall, the RDN literature reports that circulating miRNAs and circRNAs may change after renal sympathetic denervation in RH, but the evidence base is heterogeneous and small. Notably, one RDN study is available only as a congress abstract (Schirmer et al., 2013), with incomplete reporting of RH definition and specific miRNA identities, limiting interpretability. The two full-length pre–post cohorts (Dörr et al., 2016; Cai et al., 2022) observed post-procedural changes in selected miRNAs/circRNAs, but lack control groups and do not establish whether these signals are specific, reproducible, or clinically predictive. Accordingly, these findings should be interpreted as exploratory.

## 4 Discussion

Across all eight studies, the spectrum of circulating RNA biomarkers investigated in RH is fragmented, with minimal overlap in the specific miRNAs or circRNAs assessed from one study to another. The most frequently discussed candidates—serum miR-21 in baseline RH (Kara et al., 2021), miR-1-1 and miR-195 in RH with T2DM (Błaszczyk et al., 2024), the three-miRNA CPAP-response cluster (miR-378a-3p, miR-486-5p, miR-100-5p) in RH with OSA (Sánchez-de-la-Torre et al., 2015), miR-210/miR-126 in OSA screening (Novak et al., 2017), and miR-133a or selected circRNAs after RDN (Dörr et al., 2016; Cai et al., 2022)—have generally been reported in single studies and have not been replicated across independent cohorts using harmonised protocols. Importantly, three of the eight included studies were congress abstracts, including two OSA-related reports (Novak et al., 2017; Khalyfa et al., 2015), which limits methodological transparency and interpretability. No included study combined different RNA classes or integrated RNA data with genomic/epigenetic markers, and none formally evaluated the incremental value of RNA biomarkers beyond conventional clinical and biochemical predictors in robust multivariable models with external validation. Therefore, the current literature should be regarded as exploratory and hypothesis-generating, and it does not yet support claims of consistent or clinically actionable RNA “signals” in RH.

Serum miR-21 was elevated in patients with RH compared with normotensive and essential-hypertension controls in one study (Kara et al., 2021). While prior experimental work links miR-21 to RAAS/mineralocorticoid signalling and remodeling pathways (Syed et al., 2018; Ball et al., 2017; Kura et al., 2020), the RH data remain single-study and cannot establish specificity. In this context, higher circulating miR-21 in RH may reflect greater neurohormonal activation and/or a higher burden of vascular and myocardial stress rather than RH *per se*. In contrast, miR-155 did not show robust discrimination in the same study (Kara et al., 2021), despite its recognised role in vascular and immune regulation (Li et al., 2016; Zhang et al., 2024); this may indicate that its circulating signal is more closely tied to inflammatory/atherosclerotic phenotypes than to RH’s typical haemodynamic–neurohormonal profile, but this interpretation remains speculative.

In patients with RH and T2DM, distinct baseline perturbations in miR-1-1 and miR-195 were reported compared with non-diabetic RH comparators (Błaszczyk et al., 2024). Both miRNAs have been implicated in diabetic and hypertensive cardiac stress responses in experimental and clinical literature (Diao et al., 2011; Liu and Liu, 2017; Yang et al., 2024; Kura et al., 2020). However, within an RH framework, these differences may plausibly reflect comorbidity-driven cardiovascular injury and metabolic stress layered on top of hypertension, rather than an RH-specific biomarker signature.

Three miRNAs—miR-378a-3p, miR-486-5p and miR-100-5p—were reported to be associated with blood-pressure response to CPAP in men with RH and comorbid OSA, based on a small study with internal training/validation and without external replication (Sánchez-de-la-Torre et al., 2015). Experimental and non-RH clinical data support that these miRNAs participate in cardiometabolic and hypoxia-responsive pathways, although evidence is less extensive than for miR-21 or miR-155. miR-378 family members have been implicated in regulating cardiac metabolism, angiogenesis and hypertrophy, with several animal and human studies suggesting a role in limiting adverse remodelling and modulating energy homeostasis under stress conditions (Yuan et al., 2018). Circulating miR-486-5p has been linked to vascular and pulmonary-artery smooth-muscle cell proliferation, hypoxia signalling and cardiopulmonary disease; changes in miR-486-5p levels have been reported in pulmonary hypertension and myocardial infarction (Yen et al., 2022; Xu et al., 2023). miR-100-5p modulates mTOR and related growth and angiogenic pathways in vascular cells and has been studied in the context of atherosclerosis and endothelial dysfunction (Pankratz et al., 2018; Kargar et al., 2025). Collectively, these data make it plausible that a multi-miRNA signature involving miR-378a-3p, miR-486-5p and miR-100-5p could capture aspects of intermittent hypoxia, endothelial stress and sympathetic activation that determine BP responsiveness to CPAP in RH–OSA.

In hypertensive patients with suspected OSA, a panel including miR-210 and miR-126 was evaluated for non-invasive OSA screening (Novak et al., 2017). These miRNAs are well linked to hypoxia signalling and endothelial function (Wang et al., 2008; Pan et al., 2018; Egea, 2025), but the RH relevance of this abstract-only report is limited by incomplete reporting and the broader challenge that such signals may reflect OSA-related hypoxic/endothelial stress rather than RH biology.

Two studies evaluated changes in miR-133a and specific circRNAs after RDN (Dörr et al., 2016; Cai et al., 2022). miR-133a is a muscle-enriched miRNA that regulates cardiomyocyte hypertrophy, fibrosis and electrical stability, and is consistently dysregulated in cardiac hypertrophy and heart failure; experimental overexpression or inhibition of miR-133a can modulate pathological remodelling *in vivo* (Li et al., 2018). The reported RDN-associated changes in circulating miR-133a may therefore mirror evolving left-ventricular and vascular remodelling after sympathetic denervation, although the directionality and magnitude of change in the RH cohorts (Dörr et al., 2016; Cai et al., 2022) have not yet been reconciled with imaging or haemodynamic data. Beyond RH, several cardiovascular/vascular studies suggest that circRNAs can modulate key miRNAs discussed in this review, raising the possibility that analogous circRNA–miRNA modules may exist in RH and could be tested as future circulating candidates. For example, in myocardial ischemia–reperfusion models, circRNA_0031672 has been reported to interact with miR-21-5p within a circRNA_0031672/miR-21-5p/PDCD4 axis, supporting the plausibility that miR-21 signals may be embedded in broader ceRNA networks rather than reflecting a single biomarker alone (Zhang J. et al., 2021). In pulmonary vascular disease, hsa_circNFXL1_009 was computationally predicted to have a binding site for miR-210-5p, but experimental testing did not confirm effective miR-210 sponging in luciferase assays, underscoring that bioinformatic circRNA–miRNA links require rigorous validation (Jin et al., 2020). For miR-126, evidence for specific circRNA partners comes primarily from non-RH vascular/hypertensive contexts (e.g., circ_0002348/miR-126-3p reported in preeclampsia), suggesting that RH-relevant endothelial circRNAs that regulate miR-126 should be explored using unbiased profiling with paired circRNA–miRNA measurement and standardized pre-analytics (Zhou et al., 2023). Overall, these examples are intended as candidates to motivate targeted circRNA screening in RH cohorts, rather than as evidence of established circRNA–miRNA “signatures” in RH.

When viewed against the broader cardiovascular biomarker literature, the field of circulating RNAs in RH appears markedly under-developed. Systematic reviews of ncRNAs in cardiovascular disease identified hundreds diagnostic or prognostic studies encompassing acute coronary syndromes, chronic coronary artery disease, heart failure, atrial fibrillation, stroke and peripheral artery disease, and catalogued dozens of candidate ncRNAs associated with disease presence, severity or outcomes (Wang and Jin, 2020; Xie et al., 2024; Ren et al., 2025; Zhang L. et al., 2021; Yang et al., 2022; Pérez-Cremades et al., 2020).

By contrast, RH—despite its strong association with cardiovascular morbidity, mortality and therapeutic complexity—has attracted only eight ncRNA clinical studies, each probing different miRNAs or circRNAs in disparate clinical contexts. Moreover, a significant part of the hypertension-related ncRNA literature is based on tissue or cellular models rather than on circulating biomarkers, further widening the translational gap between basic discoveries and clinically deployable assays (Jusic et al., 2019).

These disparities mirror broader challenges in translating ncRNAs into clinical tools. Reviews focusing on pre-analytical and analytical issues emphasise that differences in sample type (serum vs. plasma vs. whole blood), RNA isolation methods, spike-in controls, normalisation strategies and quantification platforms can substantially affect measured miRNA levels and contribute to inconsistent results across studies (Felekkis and Papaneophytou, 2020; De Gonzalo-Calvo et al., 2022). These factors are evident in the RH literature, where studies used diverse matrices (serum, plasma, PBMC), different internal controls and non-overlapping profiling panels, complicating direct comparison and replication.

In addition, most RH studies enrolled relatively small samples (typically a few dozen patients per group) and were frequently conducted at single centres. Without careful phenotyping and larger, multi-centre cohorts, it is difficult to determine whether observed RNA signatures are specific to RH or simply reflect generic cardiovascular risk, comorbidities or prior treatment exposure.

### 4.1 Limitations of the available evidence and of this review

Several limitations of the underpinning literature substantially restrict the inferences that can be drawn from these findings:

1. Small samples and heterogeneous designs. Most included studies enrolled fewer than 100 participants, often with multiple comparison groups, limiting statistical power and increasing the risk of type I and type II errors. Designs varied widely—cross-sectional vs. longitudinal, discovery vs. candidate-driven, interventional vs. observational—further hampering synthesis.
2. Variable definitions of resistant hypertension. Definitions of RH were not uniform across studies and sometimes predated contemporary guideline criteria, with differences in required medication classes. This heterogeneity may dilute any phenotype-specific RNA signal.
3. Assay and normalisation differences. The use of different qPCR platforms, RNA extraction kits, reference miRNAs and normalisation approaches likely contributes to between-study variability and may obscure true biological associations. Standardisation efforts in the broader cardiovascular miRNA field have not yet been translated into RH research.
4. Single-marker and single-class focus. Many RH studies focused only on a small number of ncRNAs at a time. None adopted unbiased high-throughput sequencing of multiple RNA classes, and none integrated RNA markers with genomic, proteomic or metabolomic data. This narrow focus is out of step with current multi-omics approaches in other cardiovascular conditions.

A key interpretative limitation across the current RH biomarker literature is specificity. The included cohorts represent a population with a high burden of chronic kidney disease, diabetes, obesity and established cardiovascular disease, and they are typically treated with intensive multidrug regimens (including RAAS blockade, mineralocorticoid antagonists, statins and antidiabetic therapies), all of which can influence circulating ncRNA profiles. Consequently, the available data cannot determine whether the reported miRNA/circRNA patterns are RH-specific signatures (i.e., reflecting mechanisms of true treatment resistance) or instead represent more generic “high global cardiovascular risk/heavy treatment exposure” signatures that would also be present in similarly high-risk, intensively treated patients without RH. Discriminating these possibilities will require appropriately matched control groups (e.g., controlled hypertension with comparable comorbidity and treatment intensity) and robust multivariable modelling with replication.

Limitations of this scoping review include restriction to English-language publications and reliance on published data, which may introduce publication bias and underrepresent negative or inconclusive studies. Nonetheless, the scoping approach allowed us to map the breadth of circulating RNA research in RH and to identify consistent themes and gaps.

### 4.2 Future directions

Future research on circulating RNA biomarkers in RH should strive to close the gap with other cardiovascular fields in several ways. Large, multicentre cohorts of well-phenotyped RH patients with standardised diagnostic work-up, including assessment of secondary causes and medication adherence are needed to validate candidate miRNAs identified in small exploratory studies and to allow discovery of novel ncRNAs (including long ncRNAs, circRNAs or snoRNAs) using high-throughput approaches (Das et al., 2020; Zhou et al., 2018). Study designs should incorporate appropriate control groups (e.g., treated but controlled hypertension, normotension, specific comorbidity profiles) to clarify whether observed RNA signatures are truly specific to RH rather than reflecting general cardiovascular risk. Longitudinal sampling before and after therapeutic interventions (intensified pharmacotherapy, RDN, CPAP for OSA and other lifestyle or device-based strategies) would enable evaluation of dynamic biomarker changes in relation to BP response and organ damage progression.

Methodologically, adherence to best practices for pre-analytical handling, normalisation and reporting of ncRNA data will be essential to ensure comparability and reproducibility. Integration of RNA biomarkers with clinical, imaging and other omics data (e.g., proteomics, metabolomics, genomics) may help identify composite risk scores or mechanistic clusters within the heterogeneous RH population. Finally, as shown by work in other cardiovascular conditions, progress toward clinical translation will likely require not only robust observational evidence but also interventional or mechanistic studies that test whether modulating specific ncRNA pathways can favorably alter vascular or cardiac outcomes (Imbalzano et al., 2025; Oancea et al., 2025).

## 5 Conclusion

This scoping review indicates that research on circulating ncRNA biomarkers in RH remains nascent, sparse, and methodologically heterogeneous. Across eight studies—three of which were available only as congress abstracts, including two OSA-related reports—several miRNAs and circRNAs have been reported in association with specific RH phenotypes or short-term treatment response (e.g., miR-21, miR-1-1/miR-195, miR-133a, and selected circRNAs; and, in RH + OSA, a proposed multi-miRNA signature and exploratory signals for miR-210/miR-126). However, these observations are largely hypothesis-generating: many cohorts were small, analytical pipelines and normalization strategies differed substantially, most analyses were univariable, and external validation and replication across independent, well-phenotyped populations are lacking. Consequently, no circulating ncRNA biomarker can currently be recommended for routine diagnosis, risk stratification, or treatment selection in RH. Progress toward clinical translation will require adequately powered, multicentre studies with standardized assays, careful phenotyping (including adherence assessment and secondary-cause work-up), and robust multivariable modelling with external validation.

## Glossary

ABPM: ambulatory blood pressure monitoring
AHI: apnoea–hypopnoea index
ANCOVA: analysis of covariance
AUC: area under the receiver-operating characteristic curve
BMI: body mass index
BP: blood pressure
CCB: calcium-channel blocker
CPAP: continuous positive airway pressure
ddPCR: droplet digital polymerase chain reaction
GFR: glomerular filtration rate
ESC: European Society of Cardiology
ESH: European Society of Hypertension
FDR: false discovery rate
HBPM: home blood pressure monitoring
HFpEF: heart failure with preserved ejection fraction
HT: hypertension
LA: left atrium
LV: left ventricle
miRNA: microRNA
ncRNA: non-coding RNA
NRG: non-responder group
OSA: obstructive sleep apnoea
PBMC: peripheral blood mononuclear cell
PRISMA: Preferred Reporting Items for Systematic Reviews and Meta-Analyses
PRISMA-ScR: PRISMA extension for Scoping Reviews
qRT-PCR: quantitative reverse-transcription polymerase chain reaction
RAAS: renin–angiotensin–aldosterone system
RDN: renal sympathetic denervation
RG: responder group
RH: resistant hypertension
RQ: relative quantification
SBP: systolic blood pressure
T2DM: type 2 diabetes mellitus
